# Luteal phase stimulation versus follicular phase stimulation in poor ovarian responders: A systematic review and a meta‐analysis

**DOI:** 10.1002/ijgo.70883

**Published:** 2026-02-25

**Authors:** Vittorio Agrifoglio, Antonio D'Amato, Mariano Catello Di Donna, Marika Marino, Antonio Simone Laganà, Renato Venezia, Davide Vinci, Gaetano Riemma, Andrea Etrusco

**Affiliations:** ^1^ Department of Health Promotion, Mother and Child Care Internal Medicine and Medical Specialties, University of Palermo Palermo Italy; ^2^ Cliniques Universitaires Saint‐Luc Université Catholique de Louvain Brussels Belgium; ^3^ Department of Interdisciplinary Medicine, Unit of Obstetrics and Gynecology University of Bari, “Aldo Moro,” Policlinico of Bari Bari Italy; ^4^ Unit of Gynecologic Oncology National Cancer Institute Naples Italy; ^5^ Obstetrics and Gynecology, Physiopathology of Reproduction and IVF Unit, Department of Surgical Sciences Sant'Anna Hospital, University of Torino Turin Italy; ^6^ Unit of Obstetrics and Gynecology “Paolo Giaccone” Hospital Palermo Italy; ^7^ Department of Woman, Child and General and Specialized Surgery University of Campania “Luigi Vanvitelli,” Naples Italy

**Keywords:** follicular‐phase ovarian stimulation, luteal‐phase ovarian stimulation, ovarian reserve, poor ovarian responders

## Abstract

**Background:**

Poor ovarian response (POR) complicates a substantial proportion of in vitro fertilization (IVF) cycles, frequently causing cycle cancellation and suboptimal reproductive outcomes. Conventional follicular‐phase ovarian stimulation (FPOS) protocols often yield limited results, prompting the evaluation of alternative strategies such as luteal‐phase ovarian stimulation (LPOS).

**Objectives:**

This study assesses whether LPOS presents similar efficacy in terms of assisted reproductive technology (ART) outcomes compared with stimulation in the conventional follicular phase in women with POR according to Bologna criteria.

**Method:**

Comprehensive electronic searches were performed up to July 2024 across MEDLINE (via PubMed), EMBASE, SCOPUS, LILACS, SciELO, CINAHL, PsycINFO, AMED, Clinicaltrials.gov, WHO ICTRP, and Cochrane databases, with additional hand‐searching of reference lists and gray literature. Inclusion criteria were comparative studies evaluating LPOS versus FPOS in poor ovarian responders undergoing IVF/intracytoplasmic sperm injection. Studies involving DuoStim protocols, lacking control groups, or with incomplete data were excluded. Study selection, data extraction, and quality assessments (RoB 2 for randomized clinical trial (RCTs); ROBINS‐I for observational studies) were conducted independently by multiple reviewers. Meta‐analysis utilized random‐effects models to generate pooled mean differences (MD) and risk ratios (RR) with 95% confidence intervals (CI). Certainty of evidence was assessed using the GRADE methodology.

**Results:**

Five studies (312 patients; 114 LPOS vs. 198 FPOS) met the inclusion criteria. LPOS resulted in a non‐significant increase in cumulus–oocyte complexes retrieved (MD 0.56; 95% CI –0.12 to 1.23) and mature (MII) oocytes (MD 0.061; 95% CI –0.04 to 0.126), with moderate heterogeneity. The number of embryos reaching day 3 was significantly higher with LPOS (MD 0.77; 95% CI 0.29 to 1.25). No significant differences were found for clinical pregnancy rate (RR 1.26; 95% CI 0.78 to 2.02), fertilization rate (RR 1.04; 95% CI 0.91 to 1.20), pregnancy loss rate (RR 0.97; 95% CI 0.27 to 3.43), or cancellation rate (RR 0.89; 95% CI 0.34 to 2.30). Stimulation duration was significantly longer in LPOS, while total gonadotropin dose and serum estradiol on trigger day did not differ significantly. The overall certainty of evidence ranged from low to very low; therefore, findings should be interpreted as preliminary and might change as additional studies become available.

**Conclusion:**

Luteal‐phase ovarian stimulation appears to be an effective alternative to conventional FPOS for poor responders, demonstrating similar reproductive outcomes with possible quantitative benefits in embryo yield. However, the evidence is limited, and research is ongoing; therefore, results are pending and further large‐scale randomized controlled trials are required to confirm these findings.

**PROSPERO Registration:**

This review was prospectively registered in PROSPERO (ID: CRD420251010075; registration date: 12/05/2025).

## 1 INTRODUCTION

### 1.1 Background

Poor ovarian response (POR) complicates approximately 9–24% of in vitro fertilization (IVF) cycles and is responsible for more than half of cancellations, representing a critical obstacle in assisted reproduction.^1^, ^2^

The conventional approach follicular‐phase ovarian stimulation (FPOS) aims to maximize oocyte yield but exposes POR patients to supraphysiological estradiol, premature luteinization and persistently high cancellation rates despite GnRH‐antagonist co‐treatment.^3^

According to the model proposed by Baerwald et al.,^4^ for describing human ovarian follicular development, major and minor waves of follicles occur during the menstrual cycle. Therefore, the selection of a dominant follicle for preferential growth and development to an ostensibly ovulatory diameter can occur at more than one time during the same menstrual cycle.

This knowledge challenges the previously held notion that a single cohort of antral follicles grows only during the follicular phase of the menstrual cycle.^5^

The recognition that most women recruit two or three discrete follicular waves per cycle has prompted initiation of stimulation after ovulation, when progesterone physiologically suppresses LH surges.^6^ Random‐start protocols first validated for urgent onco‐fertility therefore paved the way for scheduled luteal‐phase ovarian stimulation (LPOS) in routine IVF.^7^

In poor responders, sequential follicular and luteal‐phase stimulations within the same cycle (“DuoStim”) can almost double the cumulative oocyte yield, shortening time to embryo accumulation.^8^

Nevertheless, comparative studies have reported conflicting reproductive outcomes: a recent randomized trial documented significantly higher numbers of mature oocytes and transferable embryos after LPOS,^9^ whereas another trial found clinical equivalence between LPOS and FPOS.^10^ Molecular analyses have also revealed distinct cumulus‐cell transcriptomes between protocols, suggesting potential differences in oocyte competence.^11^

### 1.2 Objectives

The objective of this systematic review and meta‐analysis was to assess whether LPOS presents similar efficacy in terms of assisted reproductive technology (ART) outcomes compared with stimulation in the conventional follicular phase in women with POR according to Bologna criteria.

## 2 MATERIALS AND METHODS

A priori definitions were made for the search approach, selection criteria, data extraction, quality assessment, and statistical analyses. The Preferred Reporting Items for Systematic reviews and Meta‐Analyses (PRISMA) standards^12^ were followed in the conduct and reporting of this systematic review and meta‐analysis, which was prospectively registered in the International Prospective Register of Systematic Reviews (ID: CRD420251010075; registration date: 12/05/2025).

### 2.1 Eligibility criteria

All the studies comparing LPOS and FPOS outcomes in women with POR undergoing ART (IVF or intracytoplasmic sperm injection [ICSI]) were included. The intervention group consisted of patients classified as POR according to Bologna criteria who underwent LPOS and then IVF or ICSI.

The control group consisted of patients classified as POR according to Bologna criteria who underwent standard FPOS and then IVF or ICSI. Studies including women that could not be classified as poor responders, that underwent a “duo‐stim” protocol, without a control (direct IVF/ICSI), and with incomplete data or lacking follow‐up records were excluded from the analysis.

### 2.2 Information sources and search strategy

The following Medical Subject Heading (MeSH) phrases and keywords were used in an unrestricted search of electronic databases, including SCOPUS, MEDLINE (available through PubMed), LILACS, EMBASE, Scielo.br: “ovarian stimulation” (MeSH Unique ID: D010062), “follicular phase” (MeSH Unique ID:D005498), “luteal phase” (MeSH Unique ID:D008183), “IVF/ICSI” (MeSH Unique IDs: D005307 and D020554), “oocyte retrieval” (MeSH Unique ID: D054315) and “reproductive outcomes.” The search string was changed to match the format of each database (Appendix S1). Further searches were conducted on CINAHL, PsycINFO, and AMED to lessen publication bias to locate more pertinent literature. Clinicaltrials.gov, the International Clinical Trials Registry Platform (ICTRP) of the World Health Organization, and the Cochrane Central Register of Controlled studies were searched to find further randomized controlled trials. Further, a search for conference abstracts from both national and international conferences was performed through the gray literature (NTIS, PsycEXTRA). The included study references and relevant reviews were also examined to find other publications that escaped our initial search. No restrictions based on language or region were applied. The analysis did not include editorials, letters to the editor, comments, and publications with second thoughts. A.E. and V.A. carried out the approach for the literature search.

### 2.3 Study selection

The titles and abstracts of all the papers were separately screened by two reviewers to identify which research should be evaluated further and to omit citations that were judged unnecessary by both observers. The authors, institutions, publication titles, and study findings were all concealed during this initial screening. Any ambiguity or disagreement was cleared up by talking with a third reviewer. Two reviewers (A.E. and G.R.) retrieved and evaluated full texts of possibly relevant articles based on the pre‐established inclusion criteria. In addition, methodological validity was evaluated before being included in the review. Every doubt or disagreement was cleared up by discussion among reviewers to reach an agreement.

### 2.4 Data extraction

Data were extracted from included papers using a data extraction form designed specifically for the review. Three authors (M.D.D., D.V., V.A.) were involved in data extraction.

The nation, study design, study type, inclusion and exclusion criteria, study period, and location, were extracted from each study. For each group (LPOS and FPOS), the following study characteristics were gathered to characterize the included studies: factors such as the number of women, the protocol for ovarian stimulation, the number of oocytes retrieved, the duration of the stimulation, the serum follicle‐stimulating hormone (FSH) levels at day 2 or 3 of the stimulation, the total dosage of FSH, the cancellation rate, the estrogen serum level on the day of the trigger, the number of retrieved and MII oocytes, the rates of fertilization and implantation, the clinical pregnancy rate (CPR), and the pregnancy loss rate (PLR). Unpublished data and clarifications were obtained by direct communication with the original study authors in cases where the study protocols reported the collection of extra outcome data or in the case of unclear items.

The meta‐analysis includes all qualifying studies with verifiable numbers for both groups and quantitative data for the co‐primary outcomes. Each outcome was examined separately according to its group allocation (FPOS, LPOS). Data analysis was conducted using Review Manager 5.3 (The Nordic Cochrane Centre, The Cochrane Collaboration, Copenhagen, 2014).

Using the Der Simonian and Laird random effects model, the summary measures were shown as the odds ratios (OR) for categorical variables and mean differences (MD) for continuous variables, along with corresponding 95% confidence intervals (CI). For every analysis, a *P*‐value (*p*) < 0.05 was deemed statistically significant.

### 2.5 Assessment of risk of bias

The methodological quality of the selected studies was assessed using the Cochrane Handbook procedures through the RoB 2 tool for randomized trials and the ROBINS‐I tool for observational studies.^13^ According to the RoB 2, in every trial that was included, seven domains were subjected to critical investigation because it was clear that these problems were associated with skewed estimations of the treatment's effects in the following ways: random sequence generation, allocation concealment, participant and staff blinding, outcome assessment blinding, insufficient outcome data, selective reporting, and other biases are the first seven factors. The evaluation of the authors' conclusions was divided into three categories: “high risk,” “low risk,” and “unclear risk” of bias.

Confounding, participant selection, intervention classification, intervention variations, missing data, outcome assessment, and choice of reported outcomes were among the ROBINS‐I instruments^14^ that were used to assess the risks of bias. Every bias criterion risk was rated as “low,” “moderate,” “serious” or “critical.”

The risk of bias evaluation was rated independently by three authors (G.R., A.E., V.A.). The debate was settled once it was concealed from A.S.L. and M.M. The certainty of evidence for each outcome was judged using the Grading of Recommendations Assessment, Development, and Evaluation working group methodology utilizing GRADEpro GDT software (GRADEpro GDT: GRADEpro Guideline Development Tool [software], McMaster University, 2015, developed by Evidence Prime).^15^

Publication bias was assessed using funnel plot analysis and Egger's test, whereas the number of studies for the outcome of interest was at least 10.^16^

### 2.6 Comparators

The following comparators were used:

*FPOS*: Patients allocated to this group underwent standard ovarian stimulation with GnRh‐antogonist protocol. Starting from day 2 or 3 of the menstrual cycle, either rFSH, hMG, or follitropin alfa was administered with or without the addition of rLH.

When the leading follicle exceeded 12–14 mm in diameter, 0.25 mg of GnRH antagonist was administered daily until the day of oocyte trigger. Either human chorionic gonadotropin (hCG) or 0.2 mg triptorelin acetate or dual trigger with rHCG plus GnRH agonist was administered when at least one dominant follicle reached 17/18 mm in diameter. Oocyte aspiration was performed under transvaginal ultrasound guidance 34–36 h after trigger.

*Luteal‐phase ovarian stimulation*: Starting between day 15 and day 18 of the menstrual cycle, either rFSH, hMG, or follitropin alfa was administered with or without the addition of rLH. Spontaneous ovulation was confirmed by transvaginal sonography and elevated progesterone level. When the leading follicle exceeded 12–14 mm in diameter, 0.25 mg of GnRH antagonist was administered daily until the day of oocyte trigger. Either hCG or 0.2 mg triptorelin acetate or dual trigger with rHCG plus GnRH agonist was administered when at least one dominant follicle reached 17/18 mm in diameter. Oocyte aspiration was performed under transvaginal ultrasound guidance 34–36 h after trigger.

### 2.7 Data synthesis

The primary outcome was the COC, defined as the mean number cumulus–oocytes complexes retrieved after an ovarian stimulation. Secondary outcomes were CPR, defined as ultrasonographic visualization of one or more intrauterine gestational sacs from at least 5 weeks of gestation; PLR, defined as a spontaneous interruption of pregnancy before 12 weeks of gestation; day 2 or 3 serum FSH of ovarian stimulation; serum estradiol level on the day of the administration of the trigger of ovulation; total dosage of FSH used; days of ovarian stimulation; number of MII oocytes; fertilization rate, defined as the number of fertilized oocytes on the total number of oocytes; the number of embryos reaching day 3 of developmental stage; and the cancellation rate, defined as the percentage of cycles that are discontinued before the egg retrieval.

## 3 RESULTS

### 3.1 Study selection

The initial search of all the electronic databases retrieved 528 results; of those, 172 were removed as duplicates. Therefore, 356 studies underwent title and abstract screening. After title and abstract screening, nine studies were selected, of which four were excluded because they did not provide interventions of interest (Appendix S2).

**FIGURE 1:**
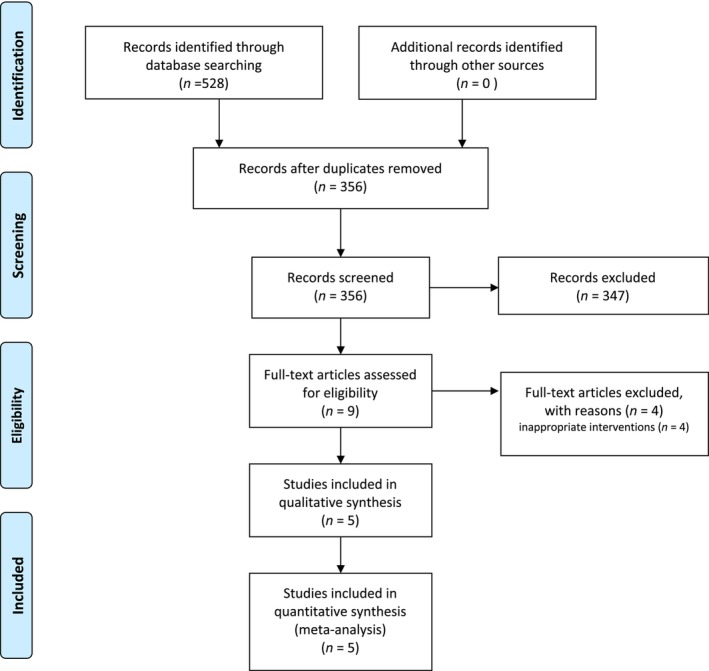
PRISMA flowchart of study selection and inclusion. *Source*: Moher et al.^17^

Five studies^2^, ^3^, ^9^, ^10^, ^11^ were finally included in the systematic review and meta‐analysis (Figure 1).

### 3.2 Study characteristics

The main characteristics of the included studies, the outcomes evaluated, and each study's inclusion and exclusion criteria are presented in Table 1.

**TABLE 1:** Characteristics of the included studies.

Author and year (study period)	Treatment	Number of patients	Study design	Main outcomes	Secondary outcomes	Inclusion criteria	Exclusion criteria	Country
Wei et al. (2016)	FPOS	123	Retrospective study	Numbers of retrieved oocytes Numbers of transferable embryos Clinical pregnancy rate	MII oocytes rate Miscarriage rate Cancellation rate Implantation rate Fertilization rate	POR according to Bologna criteria BMI between 18 and 28 kg/m2	Endometriosis Hydrosalpinx.	China
	LPOS	39						
Lin et al. (2018)	FPOS	30	Prospective cohort study	Numbers of retrieved oocytes	Number of meta‐ phase II oocytes Maturation rate Fertilization rate Number of day‐3 embryos Number of grade 1 day‐3 embryos Clinical pregnancy rate Ongoing pregnancy rate Miscarriage rate Cancellation rate	POR according to Bologna criteria	Previous oophorectomy Exposure to cytotoxic or pelvic irradiation for malignancy Positive screening for recurrent pregnancy loss Ovarian stimulating therapy during the previous 3 months Any other endocrine or metabolic disturbance	Taiwan
	LPOS	30						
Llacer et al. (2020)	FPOS	24	RCT	Number of MII oocytes	The length of stimulation FSH total dose Number of COCs obtained by follicular puncture Survival rate (after thawing) Fertilization rate	POR according to Bologna criteria Age < 41 years Regular menstrual cycles of 21–35 days Indication for starting stimulation with 300 IU of follicle‐stimulating hormone (FSH) Presence of both ovaries	Women with follicles >10 mm in the randomization visit Endometriosis stage III/IV Concurrent uterine pathology (e.g. adenomyosis, submucosal myomas, Ashermans syndrome) Concurrent participation in another study	Spain
	LPOS	24						
Chen et al. (2021)	FPOS	21	Prospective cohort study	mRNA expression of CC genes	Number of retrieved oocytes Metaphase II oocytes Fertilized oocytes, day 3 Embryos and top‐quality day‐3 embryos Biochemical pregnancy rates Clinical pregnancy rates Miscarriage rates Live birth rates	POR according to Bologna criteria two episodes of a previous POR after maximal stimulation	Diagnosis of primary ovarian insufficiency History of oophorectomy History of exposure to cytotoxic agents or pelvic irradiation for malignancy Adjuvant supplementation or hormonal replacement therapy during the previous 3 months	Taiwan
	LPOS	21						
Dastjerdi et al. (2024)	FPOS	25	RCT	Number of MII oocytes	Number of top‐quality day 3 embryo Day 3 embryo development rate Chemical pregnancy Clinical pregnancy rates	POR according to Bologna criteria undergoing FET cycle	Women with infectious diseases, sexually transmitted diseases Autoimmune disorders Tubal factor infertility, endometriosis Chronic inflammatory dis‐ eases, hormonal or anatomical disorders Endometriosis Presence of space‐occupying lesions History of ectopic pregnancy or miscarriage Myomas and polyps Previous pelvic surgeries and adhesions Cancer diagnosis Thrombophilic disorders Anemia Body mass index (BMI) ≥ 30 kg/m^2^ Chromosomal abnormalities Severe male factors	Iran
	LPOS	23						

Abbreviations: BMI, body mass index; COC, cumulus oocyte complex; FET: frozen embryo transfer; FPOS, follicular‐phase ovarian stimulation; FSH, follicle‐stimulating hormone; LPOS, luteal‐phase ovarian stimulation; POR, poor ovarian response.

Among the five included studies, two were randomized controlled trials,^9^, ^10^ two were prospective cohort studies,^3^, ^11^ and one was a retrospective cohort study.^2^ Of these, one study was from Spain,^10^ two from China,^2^, ^3^ one from Taiwan,^11^ and one from Iran.^9^ A total of 312 infertile patients undergoing ovarian stimulation were included in this analysis; of these, 198 underwent standard FPOS, and 114 patients underwent LPOS.

### 3.3 Risk of bias

The risk of bias of included studies is reported in Tables S1 and S2.

Concerning randomized clinical trial (RCTs), both studies were deemed at low risk for each evaluated item, including selection, performance, detection, attrition and reporting biases.

Regarding observational studies, according to ROBINS‐I,^14^ a study was deemed to be at overall low risk of bias.^3^ Two studies were classified as having a moderate risk of bias, principally due to moderate risk of bias in classification of interventions.^2^, ^11^ No research was judged with a critical risk of bias.

Publication bias for each outcome of interest was not evaluated because fewer than 10 studies were included in each comparison.

### 3.4 Synthesis of results

#### 3.4.1 Primary outcome

**FIGURE 2:**
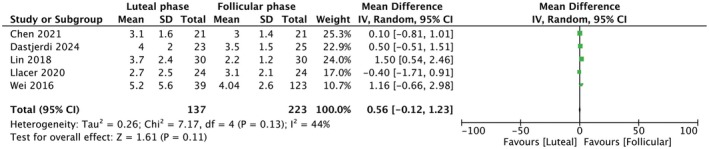
Forest plot for main outcome, number of COCs retrieved.

For the primary outcome, the number of COCs, retrieved from all five studies,^2^, ^3^, ^9^, ^10^, ^11^ no statistically significant differences were found (MD 0.56 [95% CI –0.12 to 1.23], *I* ^2^ = 44%). Only moderate heterogeneity was reported (Figure 2).

#### 3.4.2 Secondary outcomes

**FIGURE 3:**
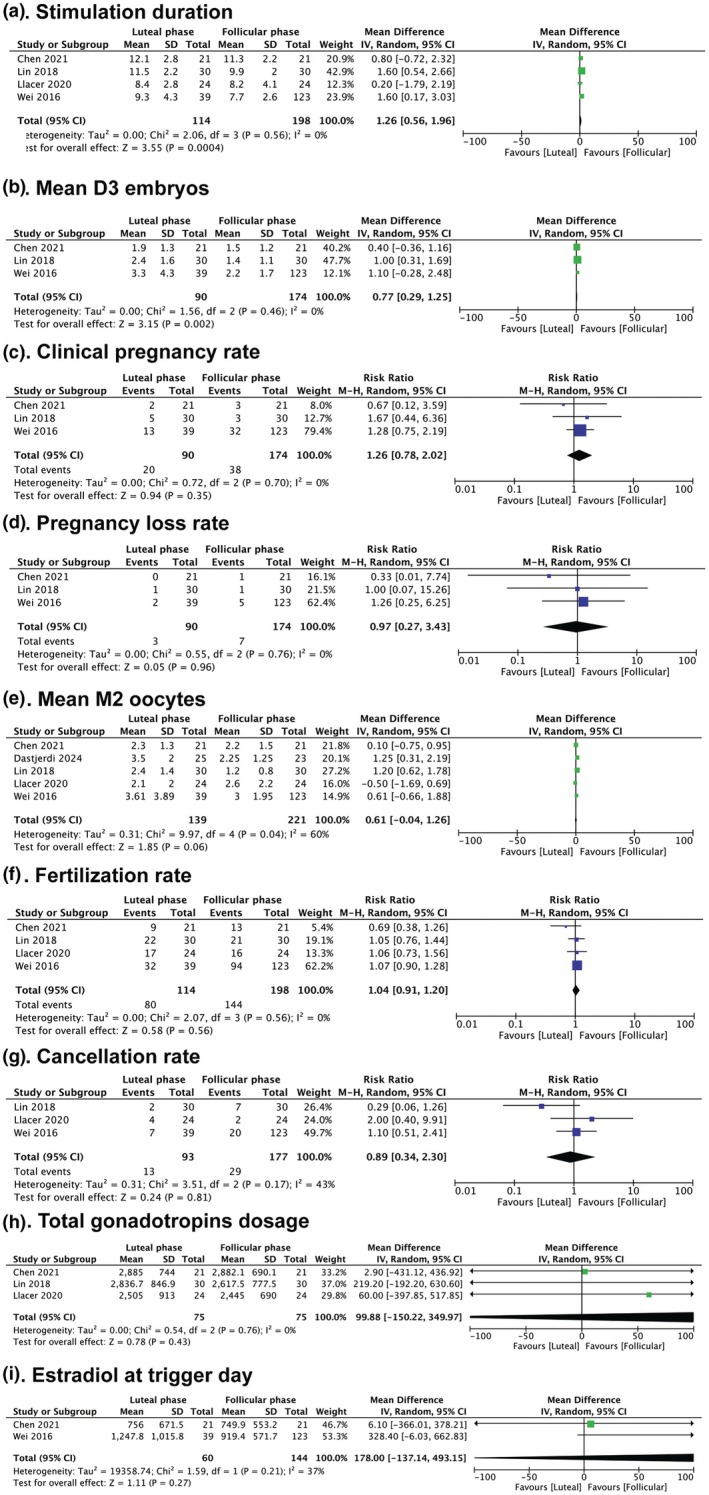
Forest plot for stimulation duration (a), mean D3 embryos (b), clinical pregnancy rate (c), pregnancy loss rate (d), mean of MII oocytes (e), fertilization rate (f), cancellation rate (g), total gonadotropins dosage (h) and estradiol at trigger date (i).

Days of ovarian stimulation were reported in four of the references.^2^, ^3^, ^10^, ^11^ The pooled estimate MD was 1.26 days (95% CI 0.56 to 1.96, *I* ^2^ = 0%), significantly longer for the LPOS group (Figure 3).

Three studies reported the mean number of embryos reaching day 3 of the developmental stage.^2^, ^3^, ^11^ The number was significantly higher in the LPOS group (MD = 0.77; 95% CI 0.29 to 1.25), with no heterogeneity detected (*I* ^2^ = 0%) (Figure 3b). Three studies reported CPR.^2^, ^3^, ^11^ The cumulative RR for this outcome was not significant (RR 1.26; 95% CI 0.78 to 2.02). There was no heterogeneity for the outcome (*I* ^2^ = 0%) (Figure 3c). PLR was evaluated in three studies.^2^, ^3^, ^11^ The weighted RR for PLR was 0.97 (95% CI 0.27 to 3.43), showing that there was no statistically significant difference between LPOS and FPOS, without heterogeneity (*I* ^2^ = 0%) (Figure 3d).

The number of MII oocytes was reported in all studies and no statistically significant difference between groups could be found (MD 0.061; 95% CI = −0.04 to 0.126). Moderate heterogeneity was reported (*I* ^2^ = 60%) (Figure 3e).

Four studies provided the fertilization rate.^2^, ^3^, ^10^, ^11^ The summary RR was 1.04 (95% CI: 0.91 to 1.20), indicating that there was no significant difference between LPOS and FPOS, with no between‐studies heterogeneity (*I* ^2^ = 0%) (Figure 3f).

The cancellation rate was reported in three studies as well and was also similar (RR = 0.89; 95% CI 0.34 to 2.30),^2^, ^3^, ^10^ with non‐statistically significant inter‐group differences and moderate heterogeneity (*I* ^2^ = 43%) (Figure 3g).

Three studies evaluated total gonadotropin dosage.^3^, ^10^, ^11^ The combined MD was not significant (99.88 IU [95% CI: −150.22–349.97], *I* ^2^ = 0%) (Figure 3h).

Two trials^2^, ^11^ assessed serum oestradiol levels on the day of the ovulation trigger. The combined MD was not significant (178.00 pg/mL [95% CI: −137.14 to 493.15]), with moderate heterogeneity (*I* ^2^ = 37%) (Figure 3i).

Certainty of evidence was judged as low for all the outcomes, except for PLR, cancellation rate and estradiol at trigger day, which were judged as very low. Detailed assessment is reported in Table 2.

**TABLE 2:** Summary of findings and certainty of evidence according to GRADE.

Outcome	No. of studies (design)	Risk of bias	Inconsistency	Indirectness	Imprecision	Publication bias	Certainty of evidence	Effect (95% CI)
COC retrieved (primary)	5 (2 RCTs, 3 OBS)	Not serious	Moderate (*I* ^2^ = 44%)	Not serious	Serious	Not assessed (*n* < 10)	⬤⬤◯◯ Low	MD 0.56 [−0.12 to 1.23]
Clinical pregnancy rate	3 (1 RCT, 2 OBS)	Not serious	Not serious	Not serious	Serious (wide CI)	Not assessed	⬤⬤◯◯ Low	RR 1.26 [0.78 to 2.02]
Pregnancy loss rate	3 (OBS)	Not serious	Not serious	Not serious	Very serious	Not assessed	⬤◯◯◯ Very low	RR 0.97 [0.27 to 3.43]
Number of MII oocytes	5 (2 RCTs, 3 OBS)	Not serious	Moderate (*I* ^2^ = 60%)	Not serious	Serious	Not assessed	⬤⬤◯◯ Low	MD 0.061 [−0.04 to 0.126]
Fertilization rate	4 (1 RCT, 3 OBS)	Not serious	Not serious	Not serious	Serious	Not assessed	⬤⬤◯◯ Low	RR 1.04 [0.91 to 1.20]
Day 3 embryos	3 (OBS)	Some concerns	Not serious	Not serious	Serious	Not assessed	⬤⬤◯◯ Low	MD 0.77 [0.29 to 1.25]
Stimulation duration	4 (2 RCTs, 2 OBS)	Not serious	Not serious	Not serious	Not serious	Not assessed	⬤⬤◯◯ Low	MD 1.26 [0.56 to 1.96]
Cancellation rate	3 (1 RCT, 2 OBS)	Not serious	Moderate (*I* ^2^ = 43%)	Not serious	Very serious	Not assessed	⬤◯◯◯ Very low	RR 0.89 [0.34 to 2.30]
Estradiol at trigger	2 (OBS)	Some concerns	Moderate (*I* ^2^ = 37%)	Not serious	Serious	Not assessed	◯◯◯ Very low	MD 178.00 [−137.14 to 493.15]

Abbreviations: CI, confidence interval; MD, mean difference; OBS, observational studies; RCT, randomized clinical trial; RR, relative risk.

## 4 DISCUSSION

### 4.1 Principal findings

This meta‐analysis confirms that LPOS in poor responders according to the Bologna criteria,^1^ represents a feasible and time‐sparing strategy in cases of poor reproductive prognosis. This quantitative analysis shows that LPOS resulted in a non‐significantly higher number of COC and MII oocytes retrieved compared to FPOS. Fertilization, CPR, and implantation and PLR rates were similar for the two protocols.

### 4.2 Comparisons with existing literature

Our results align with and expand upon findings from prior studies and reviews evaluating unconventional stimulation timing in IVF. In the past, ovarian stimulation was confined to the early follicular phase based on the classical “single follicular wave” theory, which held that a single cohort of antral follicles is recruited at the cycle's start and only that cohort can yield mature oocytes. However, recent evidence has overturned this view. It is now understood that human folliculogenesis often occurs in a wave‐like pattern, with multiple waves of follicular recruitment during the same menstrual cycle.^6^

There is also a related “continuous recruitment” theory, proposing that antral follicles are continuously entering growth phases throughout the cycle. These theories of follicular dynamics provided the physiological rationale for random‐start and luteal‐phase IVF protocols: if viable follicles exist beyond the follicular phase, then initiating stimulation at any time, even after ovulation, could help recruit those follicles for growth. Pioneering work in fertility preservation confirmed this concept. For example, Sonmezer et al.^18^ reported that starting controlled ovarian hyperstimulation in the late follicular or luteal phase for cancer patients achieved successful oocyte retrieval without delaying chemotherapy. In that case series, 9–17 oocytes were harvested from random‐start letrozole cycles initiated on cycle days 11–17, yielding 7–10 embryos – a yield comparable to conventional timing. They concluded that emergency “random‐start” stimulation (including luteal‐phase starts) is a viable approach for urgent fertility preservation, leveraging the presence of multiple follicular waves to obtain oocytes at virtually any point in the cycle. This laid the groundwork for applying LPOS in routine IVF, particularly for poor responders who might benefit from not losing time between cycles.

The studies included in our meta‐analysis directly tested the efficacy of LPOS versus FPOS in POR, and their findings are largely consistent with our aggregate results. Wei et al. conducted a retrospective study comparing a luteal GnRH antagonist protocol to a conventional follicular antagonist protocol in women with POR. They found no significant difference in the mean number of oocytes retrieved (roughly five in both groups), but notably the pregnancy rate was higher in the luteal stimulation group (46.4% vs. approximately 26% per ET). Although the analysis by Wei et al. had inherent selection biases (the luteal‐phase group had lower baseline antral follicle counts), it suggested that LPOS could achieve at least equivalent, if not superior, outcomes in a real‐world setting of poor responders.^2^ Subsequently, Lin et al. reported in a prospective pilot study of 60 POR patients that LPOS yielded significantly more oocytes and embryos than FPOS (mean approximately 3.7 vs. 2.2 oocytes) with identical protocols and patient characteristics. They also observed a higher number of metaphase II (MII) oocytes and fertilized oocytes in the luteal‐start group. Importantly, however, the clinical pregnancy rates did not differ between LPOS and FPOS in the study by Lin et al. (both around 20–30%, statistically comparable). This mirrors our finding that the benefit of LPOS is an increased oocyte/embryo yield rather than an immediate jump in pregnancy per cycle. Lin et al. hypothesized that one advantage of the luteal‐phase approach is the endocrine environment: the high progesterone levels of the luteal phase naturally prevent premature LH surges, which are a known risk in POR patients, who sometimes experience premature luteinization in follicular‐phase protocols due to reduced ovarian feedback. Thus, LPOS might offer a more controlled stimulation with a built‐in “block” against premature ovulation (because endogenous LH is suppressed by luteal progesterone), potentially improving oocyte maturation synchrony.^3^ In 2020, Llácer et al. published the first RCT on this topic, directly comparing luteal versus follicular stimulation in poor responders. Consistent with earlier retrospective studies, Llácer et al. found no statistically significant difference in the number of oocytes retrieved between LPOS and FPOS (mean MII oocytes approximately 2.1 in LPOS vs. 2.6 in FPOS, *P* = 0.31). The length of stimulation and total gonadotropin dose were similar as well. Although numerically the follicular group had a slight edge in oocyte count, the difference was not meaningful, reinforcing that luteal stimulation is non‐inferior to conventional timing in terms of ovarian response. Further, embryo metrics (i.e., number of embryos, blastocyst formation) and interim outcomes were comparable between groups. Notably, Llácer et al. performed freeze‐all cycles for both arms and thus did not report immediate fresh transfer pregnancy rates; instead, they focused on the ovarian response. Their findings support the idea that LPOS can be safely used to accumulate embryos for later transfer. The authors emphasized that their trial isolated the effect of luteal start by avoiding any preceding follicular stimulation (unlike DuoStim, where a FPOS precedes the LPOS in the same cycle). This avoids the confounding “priming” effect; that is, the possibility that an initial stimulation might enhance the ovarian responsiveness of a second stimulation.^10^ Interestingly, some reports of DuoStim noted that the second (luteal) stimulation can sometimes yield equal or more oocytes than the first. One theory is that the first stimulation effectively primes residual follicles for the subsequent LPOS, especially in a poor responder (where perhaps some follicles that did not grow in the first wave are “awakened” by the preceding gonadotropin exposure). By performing a standalone RCT of LPOS versus FPOS, Llácer et al. showed that without any follicular priming, LPOS on its own is as good as standard stimulation. However, that observation needs further validation, as their sample was small.^10^ Overall, our meta‐analysis concurs that luteal stimulation does not negatively affect outcomes and is a sound alternative schedule. More recently, Dastjerdi et al.^9^ published a single‐blinded RCT with the number of MII oocytes as the primary outcome. They found that LPOS produced significantly more mature oocytes than FPOS (mean approximately 4.5 vs. 3.2 MII, *P* < 0.01) and also a higher total oocyte count. This significant improvement in oocyte yield with LPOS is in line with the trend observed by Lin et al.^3^ and with our meta‐analysis directionality. Dastjerdi et al. did not find differences in the number of high‐quality embryos on day 3, nor in clinical pregnancy rates between the two groups, mirroring the consensus that embryo quality and implantation are equivalent between protocols. Their conclusion was that LPOS effectively increases the number of mature oocytes in women with POR without compromising embryo development. It is worth noting that all embryos in that study were frozen and transferred in subsequent cycles, as is often done for POR patients to allow cumulative embryo banking. The clinical outcomes (CPR and OPR) were low in both arms and statistically similar. Thus, consistent with the results of our meta‐analysis, Dastjerdi et al. reinforce that the advantage of LPOS is gaining more oocytes (a critical factor for poor responders, whose success often depends on numbers) while maintaining comparable pregnancy results per transfer.^9^ Chen et al. took a slightly different angle by examining the biological differences between follicular‐ and luteal‐derived oocytes. In their pilot study, they confirmed that oocyte yield and pregnancy rates were similar in the two groups but performed transcriptomic analysis on cumulus cells surrounding the oocytes. Interestingly, they found distinct gene expression profiles in cumulus cells from LPOS cycles compared to FPOS, involving genes related to inflammation, metabolism, and apoptosis. For instance, cumulus cells from LPOS showed lower expression of inflammatory cytokines (e.g., CXCL1) and certain oxidative phosphorylation genes, but higher expression of some apoptotic markers, compared to those from FPOS. The authors cautioned that these findings are preliminary and not necessarily indicative of functional differences in the oocytes; indeed, fertilization and embryo development rates were not impaired in LPOS. Nonetheless, this line of research suggests that while outward outcomes are the same, the follicular versus luteal hormonal milieu might imprint subtle molecular differences on the peri‐oocyte environment. It remains to be seen if these differences have any significance for embryo competence or if they simply reflect two paths to the same end. Importantly, the study by Chen et al. echos our results, reinforcing that from a clinical standpoint, the two protocols yield comparable results.^11^

In addition to the five studies included in our analysis,^2^, ^3^, ^9^, ^10^, ^11^ our results are supported by extensive literature on dual and alternative stimulation protocols in patients with POR and further confirm their rationale. The concept of DuoStim, introduced by Kuang et al. as the “Shanghai protocol,” showed that dual follicular and luteal stimulation in the same cycle significantly increases oocyte retrieval in patients with POR.^8^ In particular, luteal stimulation allowed for the collection of approximately twice as many oocytes as in the follicular phase, thus highlighting the effectiveness of the luteal protocol.

Subsequent studies, such as the multicenter study by Vaiarelli et al., confirmed that the luteal phase allows for a higher oocyte retrieval rate than follicular stimulation, without compromising oocyte and embryo quality, including comparable fertilization and euploid blastocyst rates.^19^ The introduction of the luteal phase significantly increased the percentage of patients who obtained at least one transferable euploid blastocyst, from 42% (follicular phase only) to 65.5% (follicular plus luteal phase), demonstrating the clinical utility of dual stimulation in maximizing oocyte and embryo yield.

Further studies have confirmed the functional equivalence of oocytes collected in the luteal and follicular phases. Martínez et al., for example, observed similar rates of oocyte maturation and pregnancy in recipients of oocytes from donors stimulated on different days of the cycle, confirming the absence of intrinsic disadvantages in luteal oocytes.^20^ Similarly, a recent multicenter analysis by Vaiarelli et al. found no significant differences in implantation, clinical pregnancy, and live births between euploid embryos derived from luteal stimulation compared to follicular stimulation. This cumulative evidence confirms the full competence of oocytes obtained in the luteal phase, further validating the results of our meta‐analysis and the clinical application of luteal stimulation in patients with reduced ovarian reserve.^21^

In summary, our findings are in concordance with the existing body of evidence: LPOS (with or without preceding follicular stimulation) is a feasible and effective strategy. The weight of the evidence suggests that while it might not dramatically improve per‐cycle pregnancy rates, it reliably increases the oocyte yield without impairing oocyte or embryo quality. The theories of multiple follicular waves provide the biological explanation, and multiple clinical studies^2^, ^3^, ^9^, ^10^, ^11^, ^22^ provide the clinical validation. Notably, a recent systematic review by Vaiarelli et al. concluded that DuoStim is a reproducible strategy to obtain more oocytes and competent embryos in a short timeframe for both fertility preservation and poor responders, while calling for further standardization of protocols and highlighting the need for individualized application.^23^ Our meta‐analysis adds quantitative reinforcement to this narrative, confirming that the luteal phase can be effectively harnessed in ART practice for women with DOR.

### 4.3 Limitations of the study

The strengths of this meta‐analysis include a focused clinical question, a rigorous methodology, and the inclusion of all the data available on this topic. We concentrated specifically on women with objectively defined diminished ovarian reserve or POR using Bologna criteria, which enhances the applicability of our conclusions to that target population. By doing so, we ensured a more homogeneous population across studies. Another strength is that our funnel plot analysis did not suggest any major publication bias, although the number of studies was small. We also assessed the quality of each study with appropriate tools according to Cochrane guidelines. Despite these strengths, there are important limitations to acknowledge. First, the number of studies and patients included is limited. Only five comparative studies met our inclusion criteria. As a result, some meta‐analytic comparisons had wide confidence intervals, and we might have been underpowered to detect small differences in some outcomes. Second, heterogeneity across studies is a concern. While all studies involved poor responders, there was variability in patient characteristics and protocols: some studies used GnRH antagonist protocols for both LPOS and FPOS,^9^, ^10^ while others used mild stimulation with clomiphene or letrozole in one or both arms.^2^, ^24^ The trigger method (hCG vs. GnRH agonist) and the decision to do fresh transfer versus freeze‐all also varied; these differences could influence outcomes. However, recognizing these limitations also offers guidance for future research: larger, multi‐center RCTs with standardized protocols are needed to confirm these outcomes and address unanswered questions (e.g., live birth benefit, optimal patient subgroup, cost‐effectiveness).

### 4.4 Implications

Luteal‐phase ovarian stimulation in women classified as POR by the Bologna criteria yields a similar number of COC and mature oocytes as FPOS, with no clinically relevant differences in clinical pregnancy, cancellation, or pregnancy‐loss rates. LPOS is therefore a safe protocol to use either alone or in the context of a DuoStim and offers a practical option when a rapid accumulation of embryos or oocytes is essential, such as in POR patients or patients with time constraints. The marginally longer stimulation and slightly higher gonadotropin consumption are not linked to measurable disadvantages.

## AUTHOR CONTRIBUTIONS

AE, VA, MM, and GR were responsible for the acquisition, analysis, and interpretation of the data. AE, AD, and VA were responsible for drafting the work. AC, CA, and DV were responsible for revising the work critically for important intellectual content. ASL, RV, MDD gave final approval of the version to be published. VA agreed to be accountable for all aspects of the work in ensuring that questions related to the accuracy or integrity of any part of the work were appropriately investigated and resolved. All authors met the International Committee of Medical Journal Editors criteria for authorship and have read and agreed to the current version of the manuscript.

## FUNDING INFORMATION

The present research was not funded.

## CONFLICT OF INTEREST STATEMENT

The authors have no conflicts of interest to declare.

## Supporting information


**Appendix S1.** Detailed search strategy for each database.

**Appendix S2.** Excluded full texts with reason for exclusion.

**Table S1.** Risk Of Bias in randomized controlled trials according to Cochrane RoB 2.0.

**Table S2.** Risk Of Bias in Non‐randomized Studies‐of Interventions (ROBINS‐I).

**Appendix S3.** PRISMA 2020 checklist.

## Data Availability

The data that support the findings of this study are available from the corresponding author upon reasonable request.
