# Supplementary material for: Luteal phase stimulation versus follicular phase stimulation in poor ovarian responders: A systematic review and a meta‐analysis
Source: Int J Gynaecol Obstet. 2026 Feb 25;174(2):608–20. doi: 10.1002/ijgo.70883 (PMC13377240; doi:10.1002/ijgo.70883)
Supplement: Supplementary file 1 — Appendix S1. Detailed search strategy for each database. [file IJGO-174-608-s001.docx]

**Appendix #1.** Detailed search strategy for each database

**Embase**

('ovarian stimulation'/exp OR 'ovarian stimulation' OR (ovarian AND ('stimulation'/exp OR stimulation))) AND ('follicular phase'/exp OR 'follicular phase' OR (follicular AND phase) OR 'luteal phase'/exp OR 'luteal phase' OR (luteal AND phase)) AND ('in vitro fertilization'/exp OR 'in vitro fertilization' OR (in AND vitro AND ('fertilization'/exp OR fertilization)) OR 'intracytoplasmic sperm injection'/exp OR 'intracytoplasmic sperm injection' OR (intracytoplasmic AND ('sperm'/exp OR sperm) AND ('injection'/exp OR injection))) AND ('oocyte retrieval'/exp OR 'oocyte retrieval' OR (('oocyte'/exp OR oocyte) AND ('retrieval'/exp OR retrieval))) AND ('reproductive outcomes' OR (reproductive AND ('outcomes'/exp OR outcomes)))

**Medline (accessed through Pubmed)**

(("ovulation induction"[MeSH Terms] OR ("ovulation"[All Fields] AND "induction"[All Fields]) OR "ovulation induction"[All Fields] OR ("ovarian"[All Fields] AND "stimulation"[All Fields]) OR "ovarian stimulation"[All Fields]) AND ("follicular phase"[MeSH Terms] OR ("follicular"[All Fields] AND "phase"[All Fields]) OR "follicular phase"[All Fields] OR ("luteal phase"[MeSH Terms] OR ("luteal"[All Fields] AND "phase"[All Fields]) OR "luteal phase"[All Fields])) AND ("in vitro fertilisation"[All Fields] OR "fertilization in vitro"[MeSH Terms] OR ("fertilization"[All Fields] AND "vitro"[All Fields]) OR "fertilization in vitro"[All Fields] OR ("vitro"[All Fields] AND "fertilization"[All Fields]) OR "in vitro fertilization"[All Fields] OR ("sperm injections, intracytoplasmic"[MeSH Terms] OR ("sperm"[All Fields] AND "injections"[All Fields] AND "intracytoplasmic"[All Fields]) OR "intracytoplasmic sperm injections"[All Fields] OR ("intracytoplasmic"[All Fields] AND "sperm"[All Fields] AND "injection"[All Fields]) OR "intracytoplasmic sperm injection"[All Fields]))) AND (("oocyte retrieval"[MeSH Terms] OR ("oocyte"[All Fields] AND "retrieval"[All Fields]) OR "oocyte retrieval"[All Fields]) AND ("reproduction"[MeSH Terms] OR "reproduction"[All Fields] OR "reproductions"[All Fields] OR "reproductive"[All Fields] OR "reproductively"[All Fields] OR "reproductives"[All Fields] OR "reproductivity"[All Fields]) AND ("outcome"[All Fields] OR "outcomes"[All Fields]))

**Scopus**

TITLE-ABS-KEY ( ovarian stimulation and ( follicular phase or luteal phase ) and ( in vitro fertilization or intracytoplasmic sperm injection ) and oocyte retrieval and reproductive outcomes )

**CENTRAL**

( ovarian stimulation and ( follicular phase or luteal phase ) and ( in vitro fertilization or intracytoplasmic sperm injection ) and oocyte retrieval and reproductive outcomes )

**CINAHL / PsycINFO / AMED / PsycExtra (accessed through EBSCO – IDEM for Italian Universities)**

( ovarian stimulation and ( follicular phase or luteal phase ) and ( in vitro fertilization or intracytoplasmic sperm injection ) and oocyte retrieval and reproductive outcomes ) Cerca anche nel testo completo degli articoli; Applica argomenti equivalenti

**Scielo.br**

( ovarian stimulation and ( follicular phase or luteal phase ) and ( in vitro fertilization or intracytoplasmic sperm injection ) and oocyte retrieval and reproductive outcomes )

**LILACS**

( ovarian stimulation and ( follicular phase or luteal phase ) and ( in vitro fertilization or intracytoplasmic sperm injection ) and oocyte retrieval and reproductive outcomes )

**Clinicaltrials.gov / ICTRP (accessed through CENTRAL)**

( ovarian stimulation and ( follicular phase or luteal phase ) and ( in vitro fertilization or intracytoplasmic sperm injection ) and oocyte retrieval and reproductive outcomes )
