# Supplementary material for: Luteal phase stimulation versus follicular phase stimulation in poor ovarian responders: A systematic review and a meta‐analysis
Source: Int J Gynaecol Obstet. 2026 Feb 25;174(2):608–20. doi: 10.1002/ijgo.70883 (PMC13377240; doi:10.1002/ijgo.70883)
Supplement: Supplementary file 2 — Appendix S2. Excluded full texts with reason for exclusion. [file IJGO-174-608-s004.docx]

**Appendix #2.** Excluded full texts with reason for exclusion

*Inappropriate interventions*

Majumdar A, Majumdar G, Tiwari N, Singh A, Gupta SM, Satwik R. Luteal Phase Stimulation in the Same Cycle Is an Effective Strategy to Rescue POSEIDON Poor Responders with No Embryos after the First Follicular Stimulation. J Hum Reprod Sci. 2023 Jul-Sep;16(3):218-226. doi: 10.4103/jhrs.jhrs_76_23.

Kuang Y, Chen Q, Hong Q, Lyu Q, Ai A, Fu Y, Shoham Z. Double stimulations during the follicular and luteal phases of poor responders in IVF/ICSI programmes (Shanghai protocol). Reprod Biomed Online. 2014 Dec;29(6):684-91. doi: 10.1016/j.rbmo.2014.08.009.

Ashrafi M, Arabipoor A, Yahyaei A, Zolfaghari Z, Ghaffari F. Does the "delayed start" protocol with gonadotropin-releasing hormone antagonist improve the pregnancy outcome in Bologna poor responders? a randomized clinical trial. Reprod Biol Endocrinol. 2018 Dec 28;16(1):124. doi: 10.1186/s12958-018-0442-y.

Madani T, Hemat M, Arabipoor A, Khodabakhshi SH, Zolfaghari Z. Double mild stimulation and egg collection in the same cycle for management of poor ovarian responders. J Gynecol Obstet Hum Reprod. 2019 May;48(5):329-333. doi: 10.1016/j.jogoh.2018.12.004.
