# Supplementary material for: Luteal phase stimulation versus follicular phase stimulation in poor ovarian responders: A systematic review and a meta‐analysis
Source: Int J Gynaecol Obstet. 2026 Feb 25;174(2):608–20. doi: 10.1002/ijgo.70883 (PMC13377240; doi:10.1002/ijgo.70883)
Supplement: Supplementary file 3 — Table S1. Risk Of Bias in randomized controlled trials according to Cochrane RoB 2.0. [file IJGO-174-608-s002.docx]

**Table S1.** Risk Of Bias in randomized controlled trials according to Cochrane RoB 2.0.

| **Study** | **Bias arising from de randomization process** | **Bias due to deviations from intended interventions** | **Bias due to missing data** | **Bias in measurement of outcomes** | **Bias in the selection of reported results** | **Overall** |
| --- | --- | --- | --- | --- | --- | --- |
| *Llacer et al. 2020* | Low | Low | Low | Low | Low | Low |
| *Dastjerdi et al. 2024* | Low | Low | Some concerns | Some concerns | Low | Low |
