# Supplementary material for: Luteal phase stimulation versus follicular phase stimulation in poor ovarian responders: A systematic review and a meta‐analysis
Source: Int J Gynaecol Obstet. 2026 Feb 25;174(2):608–20. doi: 10.1002/ijgo.70883 (PMC13377240; doi:10.1002/ijgo.70883)
Supplement: Supplementary file 4 — Table S2. Risk Of Bias in Non‐randomized Studies‐of Interventions (ROBINS‐I). [file IJGO-174-608-s003.docx]

**Table S2.** Risk Of Bias in Non-randomized Studies-of Interventions (ROBINS-I).

| **Study** | **Bias due to confounding** | **Bias in selection of partecipants into the study** | **Bias in classification of interventions** | **Bias due to deviations from intended interventions** | **Bias due to missing data** | **Bias in measurement of outcomes** | **Bias in the selection of reported results** | **Overall** |
| --- | --- | --- | --- | --- | --- | --- | --- | --- |
| *Wei et al. 2016* | Low | Some concerns | Some concerns | Low | Low | Low | Low | Some concerns |
| *Lin et al. 2018* | Low | Low | Low | Low | Low | Low | Low | Low |
| *Chen et al. 2021* | Low | Low | Some concerns | Low | Low | Low | Some concerns | Some concerns |
